# Further Evidence That the CFTR Variant c.2620-6T>C Is Benign

**DOI:** 10.1155/2017/7281023

**Published:** 2017-01-09

**Authors:** Violet I. Wallerstein, Robert Wallerstein

**Affiliations:** Hawai'i Community Genetics, Kapiolani Medical Center for Women and Children, 1319 Punahou Street, Honolulu, HI 98626, USA

## Abstract

The c.2620-6T>C variant in the* CFTR* gene is a rare variant about which little is known. We present an asymptomatic adult who has this variant as well as the well described delta F508 pathogenic variant in transpresentation. This patient provides additional evidence that this is a benign polymorphism.

## 1. Introduction

Cystic fibrosis is a genetic condition which is characterized by buildup of thick mucus affecting multiple organs, predominantly the lungs and digestive tract. The severity of the disorder varies in different individuals. Cystic fibrosis is caused by mutations in the* CFTR* gene, located at 7q31.2, that codes for a chloride ion channel. There have been more than 1800 mutations described in the* CFTR* gene. Mutations in this gene have been associated with cystic fibrosis, congenital absence of the vas deferens (CBAVD), hereditary pancreatitis, and rhinosinusitis. Different mutations have been associated with differences in severity and clinical presentation [1].

Cystic fibrosis is a relatively common genetic condition with incidence of 1 in 2,500 to 3,500 white newborns [2]. Cystic fibrosis is less common in other ethnic groups, affecting about 1 in 17,000 African Americans and 1 in 31,000 Asian Americans. Carrier frequency is 1 in 25 in non-Hispanic whites, 1 in 58 in Hispanic whites, 1 in 61 in African Americans, and 1 in 94 in Asian Americans [3]. Because of this relatively high frequency, prenatal screening programs for cystic fibrosis have been implemented in various populations around the world [4]. The American College of Obstetrics and Gynecology (ACOG) began recommending this screening in 2001 [5]. As such, pregnant women are offered carrier screening for common mutations to assess risk to have an affected infant.

## 2. Case Report

Our patient was identified through a cystic fibrosis screen performed as part of routine prenatal care. The patient was a 37-year-old African American woman who presented for prenatal care in the first trimester of pregnancy. She was in good health. She had no history of significant respiratory infections or digestive issues. Cystic fibrosis screening was offered as per routine and the patient elected this test. On that test which was a panel of 23 common mutations, she was identified to be a carrier of the delta F508 mutation. As per routine recommendations, carrier testing of her partner was discussed. At that time, her partner was not available for testing. The patient elected amniocentesis due to advanced maternal age to rule out chromosome abnormalities. She also elected testing of the amniotic fluid for cystic fibrosis. The partner was also African American. Because the detection rate in the African American population is lower and quoted as 64%, the patient elected full gene sequencing of* CFTR* since the partner was not available.

Results of testing from the amniotic fluid identified the c.2620-6T>C variant. The patient's delta F508 mutation was not found. Subsequently, the partner presented for testing. He did not carry the c.2620-6T>C variant. The patient assured us of paternity so she was tested for the c.2620-6T>C variant which she possessed. This result suggested that the delta F508 and the c.2620-6T>C variants were in transpresentation in the patient. Her older child was subsequently tested and found to have solely the delta F508 and establishing phase more strongly.

## 3. Discussion

The c.2620-6T>C variant, a T to C transition at position 2752-6 in intron 15 of the* CFTR* gene, has only been reported once in the Cystic Fibrosis Mutation Database as a splicing mutation in a patient with CBAVD, but no other symptoms were noted [6]. Our patient an adult woman who was asymptomatic also carried the pathogenic delta F508 mutation. The combination of these 2 variants in trans configuration would provide data about the pathogenic potential of the c.2620-6T>C variant. The lack of symptoms of the patient suggests that this is either a benign or very mild polymorphism. Since the patient is female, we cannot comment on potential for isolated CBAVD.

This patient provides additional information about the lack of pathogenicity of this variant. Hopefully, by reporting other patients, the true pathogenic nature of this variant can be established.
